# Agonistic Anti-TIGIT Treatment Inhibits T Cell Responses in LDLr Deficient Mice without Affecting Atherosclerotic Lesion Development

**DOI:** 10.1371/journal.pone.0083134

**Published:** 2013-12-20

**Authors:** Amanda C. Foks, Ingrid A. Ran, Vanessa Frodermann, Ilze Bot, Peter J. van Santbrink, Johan Kuiper, Gijs H. M. van Puijvelde

**Affiliations:** Division of Biopharmaceutics, Leiden University, Leiden, The Netherlands; University of Leicester, United Kingdom

## Abstract

**Objective:**

Co-stimulatory and co-inhibitory molecules are mainly expressed on T cells and antigen presenting cells and strongly orchestrate adaptive immune responses. Whereas co-stimulatory molecules enhance immune responses, signaling via co-inhibitory molecules dampens the immune system, thereby showing great therapeutic potential to prevent cardiovascular diseases. Signaling via co-inhibitory T cell immunoglobulin and ITIM domain (TIGIT) directly inhibits T cell activation and proliferation, and therefore represents a novel therapeutic candidate to specifically dampen pro-atherogenic T cell reactivity. In the present study, we used an agonistic anti-TIGIT antibody to determine the effect of excessive TIGIT-signaling on atherosclerosis.

**Methods and Results:**

TIGIT was upregulated on CD4^+^ T cells isolated from mice fed a Western-type diet in comparison with mice fed a chow diet. Agonistic anti-TIGIT suppressed T cell activation and proliferation both *in vitro* and *in vivo*. However, agonistic anti-TIGIT treatment of LDLr^−/−^ mice fed a Western-type diet for 4 or 8 weeks did not affect atherosclerotic lesion development in comparison with PBS and Armenian Hamster IgG treatment. Furthermore, elevated percentages of dendritic cells were observed in the blood and spleen of agonistic anti-TIGIT-treated mice. Additionally, these cells showed an increased activation status but decreased IL-10 production.

**Conclusions:**

Despite the inhibition of splenic T cell responses, agonistic anti-TIGIT treatment does not affect initial atherosclerosis development, possibly due to increased activity of dendritic cells.

## Introduction

Atherosclerosis, a chronic autoimmune-like disease, results from imbalanced pro- and anti-inflammatory responses, resulting in infiltration of inflammatory cells in the vessel wall. This results in the formation of an atherosclerotic plaque and eventually causes plaque rupture. Immune responses are regulated by a network of costimulatory and coinhibitory molecules present on T cells and antigen presenting cells (APCs), such as macrophages and dendritic cells. The immune system provides a large diversity of costimulatory and coinhibitory pathways and each pathway has its own unique effect on the fate of individual immune cells. Costimulatory signals can promote T cell survival, cell cycle progression and differentiation to effector and memory T cells, whereas coinhibitory molecules can terminate these processes directly or indirectly via for example the induction of regulatory T cells (Tregs).

A new-emerging complex network of costimulatory and coinhibitory molecules is formed by T cell immunoreceptor with Ig and ITIM domains (TIGIT, Vstm3, WUCAM), CD226 (DNAM-1), CD112 (PVRL2, nectin-2), and the poliovirus receptor (PVR, CD155). TIGIT is expressed on different subsets of T cells, including Tregs, activated CD4^+^ T cells, CD8^+^ T cells, and on NK cells and NKT cells. PVR is highly expressed on DCs, fibroblasts, endothelial cells and some tumor cells. [1], [2] Signaling through the TIGIT-PVR pathway can inhibit T cell responses in a cell-intrinsic manner by directly targeting the TCR signaling cascade as well as via the induction of tolerogenic DCs that produce increased levels of IL-10. [3], [4], [5] In addition, TIGIT engagement may modulate DC responses by influencing ERK activity. [5] TIGIT can also bind to CD112 present on APCs while PVR can also bind to CD226 present on T cells. TIGIT and CD226 share common binding sites on PVR and CD112 and are therefore cross-competing for binding to PVR and CD112. [3] Several studies showed that CD226 is associated with costimulatory T cell signals, as it was capable to induce Th1 responses. [6], [7], [8] Since TIGIT and CD226 compete for PVR binding, it is also believed that TIGIT attenuates T cell responses by interference of the CD226-mediated costimulation [3].

Interference in this pathway by using TIGIT deficient mice has been shown to aggravate EAE through elevated secretion of proinflammatory cytokines such as IL-6, IFN-γ and IL-17, and by increased T cell proliferation. [4] In line with this finding, Levin et al. showed that TIGIT overexpression reduces the development of EAE. [3] Furthermore, soluble TIGIT inhibits collagen-induced arthritis by dampening CD4^+^ T cell responses and by interfering with CD226-mediated costimulation. Additionally, blocking TIGIT accelerated mortality in a mouse model of graft versus host disease [3].

To date, the role of the coinhibitory TIGIT-PVR axis in atherosclerosis has not been explored. In the present study, we therefore treated LDLr^−/−^ mice with an agonistic anti-TIGIT antibody to determine the effect of coinhibitory TIGIT on atherosclerosis.

## Methods

### Animals

Female LDLr deficient (LDLr^−/−^) mice, 10–12 weeks old, were obtained from Jackson Laboratories. The animals were kept under standard laboratory conditions and were fed a normal chow diet or a Western-type diet containing 0.25% cholesterol and 15% cocoa butter (Special Diet Services, Witham, Essex, UK). Diet and water were provided ad libitum. All animal work was approved by the regulatory authority of Leiden University and carried out in compliance with the Dutch government guidelines.

### TIGIT Expression on CD4^+^ T cells under Hypercholesterolemic Conditions

Splenocytes were isolated from LDLr^−/−^ mice fed a Western-type or chow diet. Single cell suspensions were obtained by squeezing the organs through a 70 µm cell strainer. Red blood cells were removed using erythrocyte lysis buffer (0.15 M NH_4_Cl, 10 mM NaHCO_3_, 0.1 mM EDTA, pH 7.3). Subsequently, CD4^+^ T cells (>95% purity) were isolated by using the BD IMag™ mouse CD4 T lymphocyte enrichment set according to the manufacturer’s protocol (Beckton Dickinson, Mountain View, CA). After the isolation the CD4^+^ cells were stained with a fluorescent antibody for CD4 and TIGIT (clone 1G9). FACS analysis was performed on a FACSCantoII (Beckton Dickinson, Mountain View, CA). Data were analyzed using FACSDiva software (Beckton Dickinson).

### Functionality of the TIGIT Agonist under Hypercholesterolemic Conditions

Agonistic anti-TIGIT antibody was kindly provided by N. Joller. [4] To investigate the effect of agonistic anti-TIGIT on splenocyte activation and proliferation under hypercholesterolemic conditions, splenocytes from Western-type diet fed mice (n = 3) were cultured for 48 hours at 37°C in triplicate in a 96-wells round-bottom plate (2×10^5^ cells/well) in RPMI 1640 supplemented with L-Glutamine, 100 U/mL streptomycin/penicillin and 10% FCS. Splenocytes were stimulated with αCD3 and αCD28 (2 µg/mL) in the presence of different concentrations of agonistic anti-TIGIT (0–30 µg/mL) or hamster IgG (30 µg/ml). Activated T cells (CD4^+^CD25^int^ or CD4^+^CD62L^low^) were determined with FACS as described above. Proliferation was measured by addition of ^3^H-thymidine (0.5 µCi/well, Amersham Biosciences, The Netherlands) 16 hours prior to cell lysis. The amount of ^3^H-thymidine incorporation was measured using a liquid scintillation analyzer (Tri-Carb 2900R). Responses are expressed as stimulation index (SI): ratio of mean counts per minute of quintuplicate cultures with αCD3/αCD28 stimulation to quintuplicate cultures without stimulation. To detect IL-2 in the supernatant of splenocytes, an ELISA was performed according to manufacturer’s protocol (eBioscience, Vienna). Absorbance was measured at 450 nm.

### Atherosclerosis

Atherosclerosis was induced in LDLr^−/−^ mice by feeding a Western-type diet for 4 and 8 weeks. Mice were treated i.p. with 100 µg agonistic anti-TIGIT antibody (n = 9 and n = 12, respectively), 100 µg hamster IgG (Innovative Research, n = 8 and n = 12, respectively) or sterile PBS (n = 9 and n = 11, respectively) at day 0, 2, 4, 10, 17 and 24 after start of Western-type diet. At week 4 and week 8 mice were sacrificed and tissues were harvested after in situ perfusion using PBS. Tissues for histology were fixated in Zinc Formal-Fixx (Shandon Inc. Pittsburg, USA).

### Serum Cholesterol Levels

During the experiments, mice were weighed and blood samples were obtained by tail vein bleeding. The total cholesterol levels in serum were determined at week 0 and 4 after start of the 4 week experiment and at week 0, 2, 4, 6 and 8 after start of the 8 week experiment. The concentration of serum cholesterol was determined using enzymatic colorimetric procedures (Roche/Hitachi, Mannheim, Germany). Precipath (Roche/Hitachi) was used as an internal standard.

### Histological Analysis and Morphometry

Cryosections of the aortic root (10 µm) were made and stained with Oil-red-O. Lesion collagen content was determined with a Masson’s Trichrome staining. Furthermore, corresponding sections on separate slides were stained immunohistochemically with an antibody directed against a macrophage specific antigen (Moma-2, monoclonal rat IgG2b, diluted 1∶1000). Goat anti-rat IgG alkaline phosphatase conjugate (dilution 1∶100) was used as a secondary antibody and nitro blue tetrazolium and 5-bromo-4-chloro-3-indolyl phosphate as enzyme substrates. To determine the number of T cells in the lesions, a CD3 staining was performed using anti-mouse CD3 (clone SP7, dilution 1∶50, Neomarkers, Fremont, CA, USA). Morphology was studied using a Leica DM-RE microscope and LeicaQwin software (Leica imaging systems, Cambridge, UK). The percentages of collagen and macrophages in the atherosclerotic lesions were determined by dividing the area stained positive for collagen or Moma-2 by the total lesion surface area.

### Flow Cytometry

At sacrifice, blood and splenocytes were isolated (n = 5 per group). Single cell suspensions were obtained as described above. Cells were stained with fluorescent antibodies for CD11c, MHC-II and CD40 to detect dendritic cells and to determine their activation status. In a separate in vitro experiment, DCs were co-cultured with CD4^+^ T cells in a 1∶4 ratio and increasing concentrations of agonistic anti-TIGIT. After 48 hours, proliferation of T cells was observed by ^3^H-thymidine incorporation and DCs and T cells were separated from each other and stained with fluorescent antibodies for CD11c/IL-10 and CD4/CD62L respectively.

### Spleen Cell Proliferation

At sacrifice, splenocytes (n = 5 per group) were cultured for 72 hours in quintuplicate in a 96-wells round-bottom plate (2×10^5^ cells/well) in RPMI 1640 supplemented with L-Glutamine, 100 U/ml streptomycin/penicillin and 10% FCS. As a positive control cells were stimulated with αCD3 and αCD28 (2 µg/ml). Proliferation was measured as described above.

### Statistical Analysis

All data are expressed as mean±SEM. A one-way ANOVA with post test was performed to compare normally distributed data between three groups of animals. Probability values of *P*<0.05 were considered significant.

## Results

### TIGIT is Upregulated on CD4^+^ T cells from Western-type Diet Fed LDLr^−/−^ Mice

**Figure 1:**
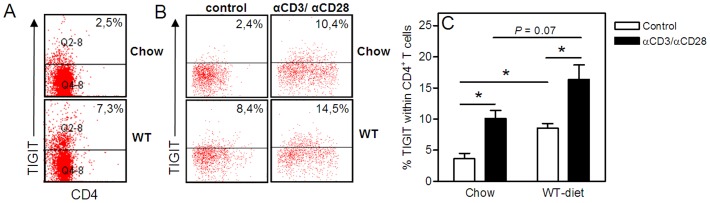
Upregulation of TIGIT expression on CD4^+^ T cells under hypercholesterolemic conditions. Representative FACS dot plots of TIGIT surface expression on CD4^+^ T cells isolated from LDLr^−/−^ mice fed a Chow diet or a Western-type (WT) diet (A). Splenocytes from LDLr^−/−^ mice fed a Chow diet (n = 3) and Western-type diet (n = 3) were cultured for 48 hours in the presence or absence of anti-CD3/anti-CD28. Representative dot plots (B) and the mean percentage of TIGIT expressing CD4^+^ T cells in 4 different conditions (C) were obtained by flow cytometry. *P<0.05.

The coinhibitory molecule TIGIT is mainly expressed on T cells and previous studies have shown that upon TCR stimulation the number of TIGIT expressing CD4^+^ T cells increases. [3], [4], [5] To investigate the effect of a high-fat diet on the surface expression of TIGIT on CD4^+^ T cells, we isolated CD4^+^ T cells from spleens of LDLr^−/−^ mice fed a chow diet or a cholesterol-rich Western-type (WT) diet and performed a FACS analysis to determine TIGIT expression. Upon WT diet feeding we observed an increase in TIGIT-expressing CD4^+^ T cells when compared with chow diet feeding (7,3% vs. 2,5%, respectively, Figure 1A). In addition, we isolated splenocytes from chow diet fed mice (n = 3) and Western-type diet fed mice (n = 3) and cultured them for 48 hours in the presence or absence of αCD3/αCD28 stimulation. As shown in Figure 1B and 1C, Western-type diet feeding significantly enhanced the percentage of TIGIT^+^ cells within the unstimulated CD4^+^ T cell population (8.5±0.7%) in comparison with chow diet feeding (3.7±0.9%, *P*<0.05). In addition, we show that αCD3/αCD28 stimulation indeed increased the percentage of TIGIT^+^ cells within the CD4^+^ T cells in both chow (10.1±1.3%, *P*<0.05) and Western-type diet mice (16.4±2.3%, *P*<0.05).

### Agonistic Anti-TIGIT Inhibits the Activation and Proliferation of Splenocytes

**Figure 2:**
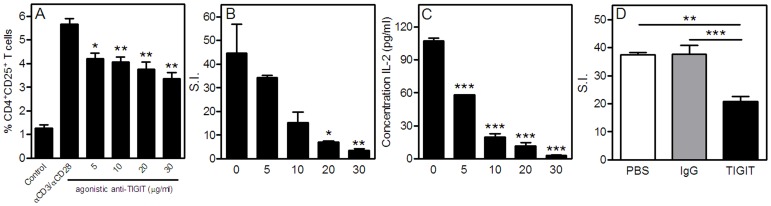
Agonistic anti-TIGIT strongly inhibits T cell function. Splenocytes from Western-type diet fed mice (n = 3) were cultured for 72 hours with αCD3/αCD28 in the presence or absence of agonistic anti-TIGIT (0–30 µg/ml). Activated T cells (CD4^+^CD25^+^) were determined with flow cytometry (A). Proliferation was assessed by the amount of ^3^H-thymidine incorporation in dividing cells and is expressed as stimulation index (B) and by the amount of IL-2 produced by the splenocytes as determined with ELISA (C). Splenocytes of PBS, Armenian Hamster IgG and agonistic anti-TIGIT-treated mice (n = 5 per group) were cultured in the presence of αCD3/αCD28 stimulation and proliferation was assessed by the amount of 3H-thymidine incorporation expressed as stimulation index (D). *P<0.05, **P<0.01, ***P<0.001.

Since TIGIT can directly inhibit T cell proliferation, we determined ex vivo the capacity of the agonistic anti-TIGIT antibody to impair T cell reactivity in our atherosclerosis mouse model; LDLr^−/−^ mice fed a Western-type diet. We stimulated splenocytes from Western-type diet fed mice for 72 hours with αCD3/αCD28 in the presence or absence of different concentrations of agonistic anti-TIGIT. As shown in Figure 2A and Figure S1A, agonistic anti-TIGIT decreased the percentage of activated T cells (*P*<0.01). Most importantly, splenocyte proliferation as measured by the amount of ^3^H-thymidine incorporation (Figure 2B and Figure S1B) and IL-2 secretion (Figure 2C), was strongly inhibited in a dose-dependent manner upon excessive TIGIT triggering when compared with the control condition (no IgG) or Armenian hamster IgG.

### Impaired T cell Function in Agonistic Anti-TIGIT-treated Mice

To study the effect of TIGIT triggering on T cells *in vivo*, LDLr^−/−^ mice (n = 9) were treated with an agonistic anti-TIGIT antibody at day 0, 2, 4, 10, 17 and 24, while being fed a Western-type diet for 4 weeks. As a control, mice were treated with Armenian hamster IgG (n = 8) and PBS (n = 9). To determine the proliferative capacity of T cells from agonistic anti-TIGIT-treated mice in comparison with PBS and Armenian hamster IgG-treated mice, splenocytes from all groups were cultured for 72 hours in the presence of αCD3/αCD28. A significant 45% decrease in splenocyte proliferation was observed in mice treated *in vivo* with agonistic anti-TIGIT (stimulation index of 20.8±1.8) compared with PBS mice (stimulation index of 37.4±0.8, *P*<0.01) and hamster IgG mice (stimulation index of 38.2±3.2, *P*<0.001, Figure 2D).

### Agonistic Anti-TIGIT Treatment does not Affect Initial Atherosclerosis Development

**Figure 3:**
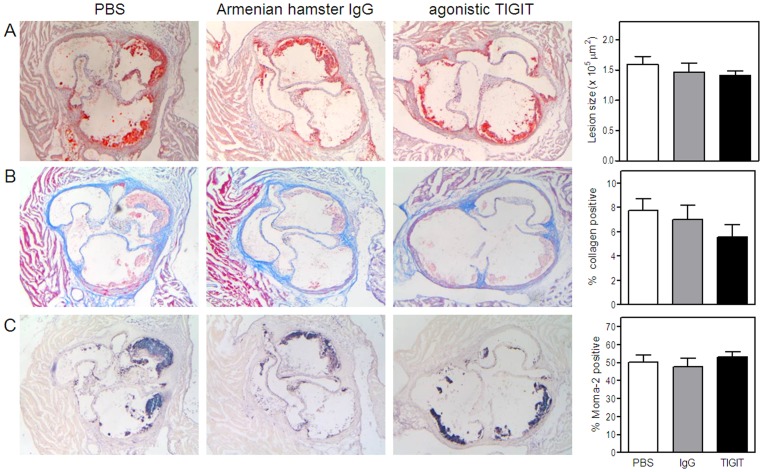
Agonistic anti-TIGIT treatment does not reduce initial atherosclerotic lesion development. No difference in atherosclerotic lesion size between agonistic anti-TIGIT, Armenian Hamster IgG and PBS treated LDLr^−/−^ mice fed a Western-type diet for 4 weeks. Representative cross-sections of lesion formation in the three valves area of the aortic root stained with Oil-Red-O and hematoxylin are shown and lesion size was determined (A). Sections of the aortic root were stained for collagen using Masson’s trichrome staining. The percentage of collagen relative to the lesion size was determined (B). Furthermore, relative macrophage content was determined with a Moma-2 staining and quantified (C).

To determine whether the TIGIT-mediated impairment of T cell function affects the development of early atherosclerosis (4 weeks of Western type diet), we measured atherosclerotic lesion sizes upon agonistic anti-TIGIT treatment. As shown in Figure 3A, no difference in atherosclerotic lesion size was observed after agonistic anti-TIGIT treatment (1.41±0.07×10^5^ µm^2^) in comparison with Armenian hamster IgG treatment (1.46±0.15×10^5^ µm^2^) or PBS (1.59±0.13×10^5^ µm^2^). During the experiment, agonistic anti-TIGIT treatment did not affect body weight and total plasma cholesterol levels (data not shown). Furthermore, collagen content did not significantly differ between the three groups (PBS: 7.7±0.9%, Armenian hamster IgG: 7.0±1.2% and TIGIT: 5.5±1.0%, Figure 3B). In addition, the percentage of macrophages in the lesions is comparable in all the groups (PBS: 50.3±4.2%, Armenian hamster IgG: 47.7±4.9% and agonistic anti-TIGIT: 53.2±3.5%, Figure 3C).

### Increased Percentages and Activation of Dendritic Cells in Agonistic Anti-TIGIT-treated Mice

**Figure 4:**
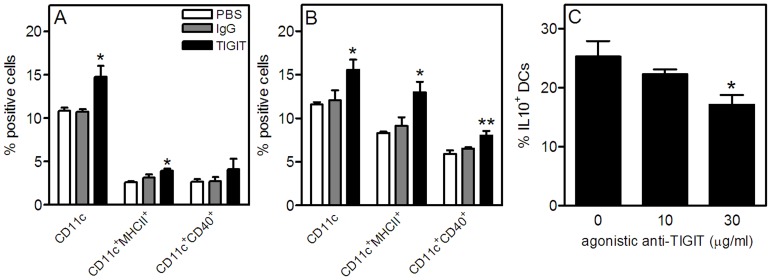
Enhanced dendritic cell percentages and activation and decreased IL-10 expressing dendritic cells after agonistic anti-TIGIT treatment. At sacrifice, blood (A) and spleen (B) cells were isolated and stained for dendritic cells and activation markers and analyzed by flow cytometry (n = 5 per group). The effect of agonistic anti-TIGIT on IL-10 expression by DCs was determined by culturing splenocytes with increasing concentrations of agonistic anti-TIGIT (C). *P<0.05, **P<0.01.

Whereas agonistic anti-TIGIT treatment reduced T cell proliferation, dendritic cells which are also involved in the pathogenesis of atherosclerosis were elevated in these mice. As shown in Figure 4A, agonistic anti-TIGIT treatment significantly enhanced the percentage of dendritic cells (14.7±1.3%, *P*<0.05) in the blood in comparison with Armenian hamster IgG and PBS treatment (10.7±0.4% and 10.8±0.4%, respectively). Similar results were observed in the spleen (Figure 4B), where agonistic anti-TIGIT treatment enhanced the percentage of dendritic cells (15.6±1.2%, *P*<0.05) in comparison with Armenian hamster IgG and PBS treatment (12.0±1.2 and 11.6±0.3%, respectively). Moreover, their activation status as measured with MHCII and CD40 is also elevated in agonistic anti-TIGIT-treated mice compared with both control groups. On the other hand, we observed a decrease in IL-10 expressing tolerogenic DCs when splenocytes were cultured with increasing concentrations of agonistic anti-TIGIT suggesting that the agonistic anti-TIGIT treatment blocked the normal interaction between TIGIT and PVR (*P*<0.05, Figure 4C).

### Agonistic Anti-TIGIT Treatment does not Affect Atherosclerosis after 8 Weeks of Western-type Diet

**Figure 5:**
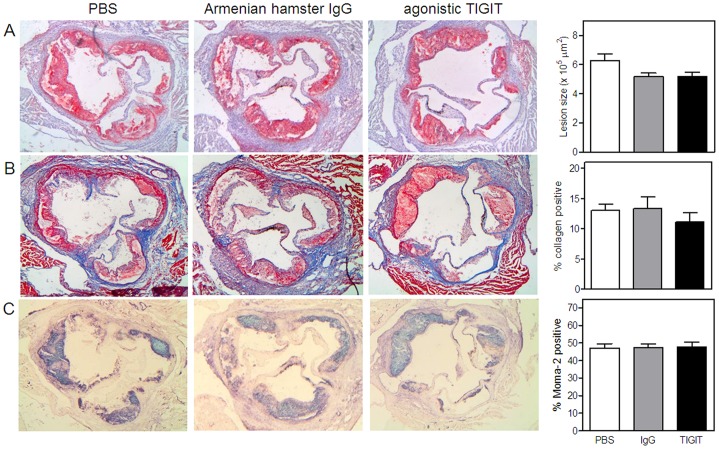
Agonistic anti-TIGIT treatment does not reduce more advanced atherosclerosis. Agonistic anti-TIGIT treatment (n = 12) and Armenian Hamster IgG treatment (n = 11) reduces atherosclerosis development in LDLr^−/−^ mice fed a Western-type diet for 8 weeks in comparison with PBS treatment (n = 12). Representative cross-sections of lesion formation in the three valves area of the aortic root stained with Oil-Red-O and hematoxylin are shown and lesion size was determined (A). Sections of the aortic root were stained for collagen using Masson’s trichrome staining. The percentage of collagen relative to the lesion size was determined (B). Furthermore, relative macrophage content was determined with a Moma-2 staining and quantified (C).

We also determined more advanced atherosclerotic lesion development after 8 weeks of Western-type diet feeding in combination with agonistic anti-TIGIT treatment during the first 24 days. Lesion size in the agonistic anti-TIGIT-treated mice (n = 12, 5.15±0.32×10^5^ µm^2^) was not affected compared with lesion size in the Armenian hamster IgG-treated mice (n = 11, 5.12±0.26×10^5^ µm^2^). In fact, both groups of antibody-treated mice have 18% smaller atherosclerotic lesions compared with the PBS group (n = 11, 6.28±0.44×10^5^ µm^2^) although this did not reach statistic significance (Figure 5A). Furthermore, no differences were observed in collagen content (PBS: 13.1±1.0%, hamster IgG: 13.3±2.0% and agonistic anti-TIGIT: 11.1±1.5%, Figure 5B) and macrophage content (PBS: 47.2±2.3%, IgG: 47.4±1.9% and agonistic anti-TIGIT: 47.9±2.6%, Figure 5C) of the atherosclerotic lesions and although agonistic anti-TIGIT reduced proliferation of T cells in the spleen, no effect was observed on intimal and perivascular CD3^+^ T cells upon agonistic anti-TIGIT treatment (Figure S2).

## Discussion

The TIGIT/CD226 pathway has been associated with several human autoimmune diseases [8] and studies in mice demonstrate that interference in this pathway may be an attractive approach to modulate autoimmune diseases. [3], [4], [5] In the present study we determined the role of TIGIT in atherosclerosis.

Previous studies have shown that TIGIT is mainly expressed on activated CD4^+^ T cells in both men and mice. [4], [9] We have previously shown that during the induction of atherosclerosis by feeding a Western-type diet, T cells are activated. [10] We now observe that TIGIT expression was upregulated on CD4^+^ T cells in Western-type diet fed LDLr^−/−^ mice in comparison with chow diet fed LDLr^−/−^ mice. The expression of TIGIT was further enhanced after αCD3/αCD28 stimulation of isolated splenic T cells. This increase in TIGIT surface expression has been associated with a decrease in T cell proliferation in a number of studies [4], [9], while TIGIT deficiency remarkably increased T cell proliferation in lymph nodes and spleen upon immunization. [4] Since T cell activation is strongly correlated with the development of atherosclerosis [11], [12], [13], we aimed to diminish T cell responses by using an agonistic anti-TIGIT antibody which triggers TIGIT signaling. First we showed that exposure of splenocytes isolated from Western-type diet fed mice to agonistic anti-TIGIT *ex vivo* greatly inhibited T cell activation and proliferation, as measured by ^3^H-thymidine incorporation and IL-2 secretion. In agreement with our *in vitro* data, LDLr^−/−^ mice that received Western-type diet for 4 weeks and were treated with the agonistic anti-TIGIT antibody, show a 45% decrease in splenocyte proliferation in comparison with PBS and Hamster IgG-treated mice.

Subsequently, we investigated whether agonistic anti-TIGIT treatment can be beneficial for the development of atherosclerosis since TIGIT-mediated dampening of T cell responses has been associated with decreased susceptibility to several autoimmune diseases. Levin et al. showed that administration of soluble TIGIT inhibited the severity of collagen-induced arthritis by decreasing T cell infiltration in the paws and by reducing T cell proliferation. [5] Interestingly, both pro-inflammatory cytokines such as IL-6, IL-17A and TNF-α, and anti-inflammatory cytokines such as IL-10 were reduced in soluble TIGIT-treated mice. Furthermore, TIGIT transgenic mice are protected against the development of EAE [5], whereas TIGIT^−/−^ mice develop exacerbated EAE through elevated T cell proliferation and increased IL-6, IFN-γ, and IL-17 secretion. [4] In addition, adoptive transfer of TIGIT-deficient T cells accelerated GVHD in comparison with transfer of wild-type T cells. [5] Surprisingly, the significant effect of the TIGIT agonist on splenic T cell responses did not affect the development of early and more advanced atherosclerosis (4 and 8 weeks of Western-type diet feeding respectively), as we observed no significant differences in atherosclerotic lesion sizes between PBS, Armenian hamster IgG and agonistic anti-TIGIT-treated mice. Furthermore, in both atherosclerosis studies we did not observe any differences in collagen, macrophage and T cell content of these lesions.

Interestingly, the beneficial effect of the TIGIT agonist on splenic T cell activity was accompanied by an activating effect on DCs. Dendritic cells are potent antigen presenting cells and numerous studies have shown the importance of DCs in the development of atherosclerosis. The number of DCs increases with the progression of atherosclerosis in ApoE^−/−^ mice [14], [15] and Wu et al. showed that CD11c^−/−^ApoE^−/−^ mice fed a Western-type diet have reduced atherosclerosis with a concomitant attenuation of lesional macrophages. [16] Additionally, Paulson et al. showed that CD11c-diphtheria toxin receptor (DTR) LDLr^−/−^ mice fed a cholesterol-rich diet for 5–10 days have a 55% reduced intimal lipid area in comparison with non-depleted mice. [17] Therefore, increased percentages and activation of dendritic cells in agonistic anti-TIGIT-treated mice can possibly counter-act the diminished T cell activity in these mice and thereby neutralize the effect on atherosclerosis. This more pro-inflammatory phenotype of DCs in agonistic anti-TIGIT-treated mice may be caused by the agonistic antibody which blocks the normal interaction between TIGIT and PVR expressed on DCs normally resulting in a tolerogenic phenotype of DCs. [5] This is confirmed in the present study by the decrease in IL-10 producing tolerogenic DCs after culturing splenocytes with increasing concentrations of agonistic anti-TIGIT.

In conclusion, we showed that although triggering of the TIGIT pathway decreases proliferation and activation of splenic T cells both in vitro and in vivo, it does not affect atherosclerosis development and local T cell numbers. Future research should concentrate more on the role of TIGIT-PVR signaling, since the generation of tolerogenic DCs in combination with intrinsic T cell inhibition possibly does affect atherosclerosis.

## Supporting Information

Figure S1 **Agonistic anti-TIGIT strongly inhibits T cell function.** DCs and CD4^+^ T cells were isolated from Western-type diet fed mice (n = 3) and were co-cultured in a 1∶4 ratio for 48 hours with αCD3/αCD28 in the presence of agonistic anti-TIGIT (30 µg/ml) or Armenian Hamster IgG (30 µg/ml). Activated T cells (CD4^+^CD62L^low^) were determined with flow cytometry (A). Proliferation was assessed by the amount of ^3^H-thymidine incorporation in dividing T cells and is expressed as stimulation index (B). *P<0.05, ***P<0.001.
(TIF)

Figure S2 **Agonistic anti-TIGIT treatment does not affect CD3^+^ T cell numbers in atherosclerotic lesions.** LDLr^−/−^ mice fed a Western-type diet for 8 weeks were treated intraperitoneally with PBS Armenian Hamster IgG or agonistic anti-TIGIT. Representative cross-sections of lesion formation in the three valves area of the aortic root were stained with anti-CD3 (A) to analyze effects on T cells in the intima (B) and perivascular tissue (C) of atherosclerotic lesions.
(TIF)
