# Supplementary figures and images for: Agonistic Anti-TIGIT Treatment Inhibits T Cell Responses in LDLr Deficient Mice without Affecting Atherosclerotic Lesion Development
Source: PLoS One. 2013 Dec 20;8(12):e83134. doi: 10.1371/journal.pone.0083134 (PMC3869776; doi:10.1371/journal.pone.0083134)

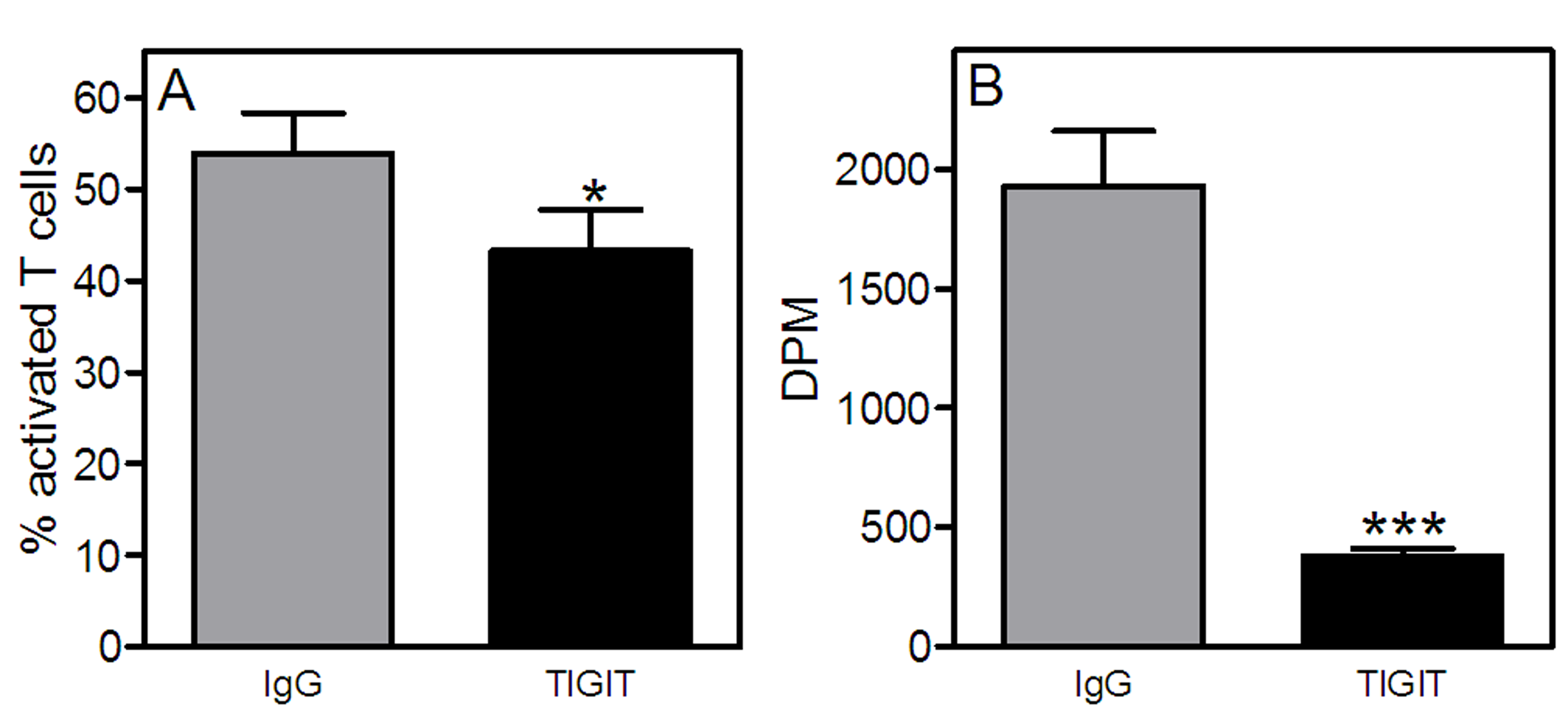

Supplement: Figure S1 — Agonistic anti-TIGIT strongly inhibits T cell function. DCs and CD4+ T cells were isolated from Western-type diet fed mice (n = 3) and were co-cultured in a 1∶4 ratio for 48 hours with αCD3/αCD28 in the presence of agonistic anti-TIGIT (30 µg/ml) or Armenian Hamster IgG (30 µg/ml). Activated T cells (CD4+CD62Llow) were determined with flow cytometry (A). Proliferation was assessed by the amount of 3H-thymidine incorporation in dividing T cells and is expressed as stimulation index (B). *P<0.05, ***P<0.001. (TIF) [file pone.0083134.s001.tif]

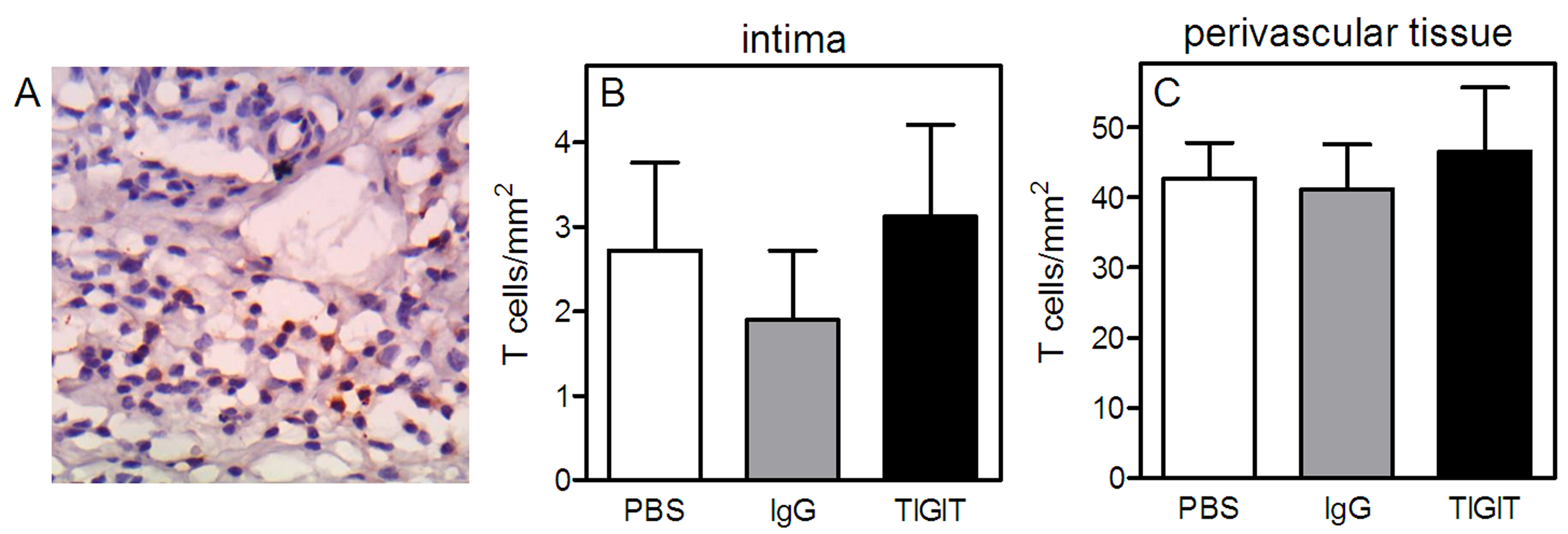

Supplement: Figure S2 — Agonistic anti-TIGIT treatment does not affect CD3+ T cell numbers in atherosclerotic lesions. LDLr−/− mice fed a Western-type diet for 8 weeks were treated intraperitoneally with PBS Armenian Hamster IgG or agonistic anti-TIGIT. Representative cross-sections of lesion formation in the three valves area of the aortic root were stained with anti-CD3 (A) to analyze effects on T cells in the intima (B) and perivascular tissue (C) of atherosclerotic lesions. (TIF) [file pone.0083134.s002.tif]
